# Chlamydia infection, PID, and infertility: further evidence from a case–control study in China

**DOI:** 10.1186/s12905-022-01874-z

**Published:** 2022-07-15

**Authors:** Lijun Liu, Changchang Li, Xuewan Sun, Jie Liu, Hepeng Zheng, Bin Yang, Weiming Tang, Cheng Wang

**Affiliations:** 1 Dermatology Hospital of Southern Medical University, Guangzhou, 510095 China; 2 Guangdong Provincial Center for Skin Diseases and STIs Control, Guangzhou, 510095 China; 3 Southern Medical University Institute for Global Health, Guangzhou, 510095 China; 4 University of North Carolina Project-China, Guangzhou, 510095 China; 5 Guangzhou Key Laboratory for Sexually Transmitted Diseases Control, Dermatology Hospital of Southern Medical University, Guangzhou, 510095 China

**Keywords:** Chlamydia, Pelvic inflammatory disease, Infertility, Women, China

## Abstract

**Background:**

*Chlamydia trachomatis* infection and pelvic inflammatory disease (PID) are well-known risk factors for female infertility. But there is limited evidence from China. This study aimed to further explore the associations between previous/current chlamydial infection, PID, and infertility in China.

**Methods:**

We performed a 1:2 matched case–control study with two control groups: pregnant controls and non-pregnant controls in China in 2019. Women diagnosed with infertility were selected as cases (n = 255). Controls were selected based on the following criteria: Pregnant women who were documented in the selected hospitals were chosen as Pregnant controls (n = 510), and people who sought health care in Obstetric/Gynecologic clinics, Family Planning clinics, Dermatology and STD Department or Urological department were selected as Non-pregnant controls (n = 510)**.** Infertility induced by male factors and people who used antibiotics in the vagina within two weeks were excluded. The first-stream specimen of urine samples was tested for *chlamydia* by nucleic acid amplification testing (NAAT). Conditional logistic regression was used to estimate the association.

**Results:**

The prevalence of previous chlamydial infection and PID were significantly higher in cases (2.4%, 17.3%) than in controls (Non-pregnancy: 0.4%, 3.0%; Pregnancy: 0.4%, 9.0%). The current chlamydial infection rates were 5.9%, 7.3%, and 7.1% in infertile, pregnant, and non-pregnant women, respectively. After adjusting for confounders, PID largely elevated the risk of infertility (using non-pregnant controls: adjusted OR = 2.57, 95% CI 1.51, 4.39; using pregnant controls: adjusted OR = 6.83, 95% CI 3.47, 13.43). And the positive association between PID and tubal infertility was more obvious for both groups. For current chlamydial infection, none of the odds ratios were significant at the 0.05 level, while small sample size limited the evaluation of an association between prior chlamydial infection with infertility.

**Conclusions:**

Previous PID was indicated to largely increase the risk of infertility, especially tubal infertility. And there should be continuing emphasis on highly sensitive and specific biomarker for prior chlamydial infection.

**Supplementary Information:**

The online version contains supplementary material available at 10.1186/s12905-022-01874-z.

## Background

Infertility refers to the failure to achieve a clinical pregnancy after over one year of regular unprotected sexual intercourse [1]. Infertility is a highly prevalent global condition and the incidence is on the rise, which is estimated to affect around 9% of reproductive-aged couples worldwide [2]. In China, the overall prevalence of infertility has reached 15.5%, while among women actively trying to conceive, the prevalence is as high as 25% [3]. Quite a few studies have revealed a negative impact of infertility on endometrial, myometrial, cervical, and placental alterations that underlie the poor obstetric outcomes [4]. It not only creates a considerable cost burden but also introduces tensions to the family. Further insight into the causes is critical to help alleviate the burden.

*Chlamydia trachomatis* is one of the most prevalent sexually transmitted microorganisms. Ascending from the lower genital tract, chlamydial infection can lead to serious reproductive consequences including infertility [5]. It was estimated that the proportion of tubal infertility caused by chlamydia had reached 45% [5]. Furthermore, this sexually transmitted microorganism is an important cause of pelvic inflammatory disease (PID), and PID was also indicated to increase the risk of infertility, then PID represents the link between chlamydia infection and infertility [6–8]. However, despite numerous studies identifying chlamydial infertility, the true host and pathogen determinants underlying infertility remain unknown due to the lack of appropriate experimental models and suitable diagnostic tools [5, 9], and the current evidence base was weak for several reasons. First, the poor performance of serological tests that were commonly used in those studies [5]. Second, pregnant women were commonly selected as control participants, of whom are different from the source population and generally exaggerate the association [10].

China provided a great opportunity to further explore the association between chlamydial infection, PID and infertility for the following reasons. First, China has a considerable disease burden of chlamydial infection. According to the National Notifiable Infectious Disease Reporting System, the reported annual incidence rate in females was 84.55 per 100,000 population in 2019, which was highly underestimated due to the insufficient chlamydia screening/testing and poor detection methods [11]. Second, the implementation of the universal two-child policy in recent years encouraged many people to seek care for infertility, which increased our access to infertile women.

In this case–control study, we aimed to investigate whether previous/current chlamydial infection and previous PID would influence the risk of infertility in the Chinese population.

## Methods

### Study population

This was a 1:2 matched case–control study aiming to explore the effects of chlamydial infection and PID on infertility. Women aged between 18 to 44 years were considered for the study if they sought for health care between Jan 1, 2019, and Oct 30, 2019, at outpatient settings, including clinics or auxiliary reproductive center, in five cities (Guangzhou, Zhanjiang, Shenzhen, Dongguan, Yunfu) of Guangdong Province, China [12].

Infertility was defined as failure to conceive after 12 months or more of regular unprotected sexual intercourse [1]. All cases involved in our study underwent a diagnostic workup by professionals, and if the patients self-reported infertility, they would receive laparoscopy and hysterosalpinography for further verification. And according to the American Society for Reproductive Medicine, we further classified infertility into several subtypes, including tubal disorders, endocrine disturbance, cervical/uterine/peritoneal factors, immune factors, sex factors, and others or unknown [13]. The participants were excluded if they met one of the following conditions: (1) infertility induced by male factors, and (2) who used antibiotics in the vagina within two weeks.

**Fig. 1:**
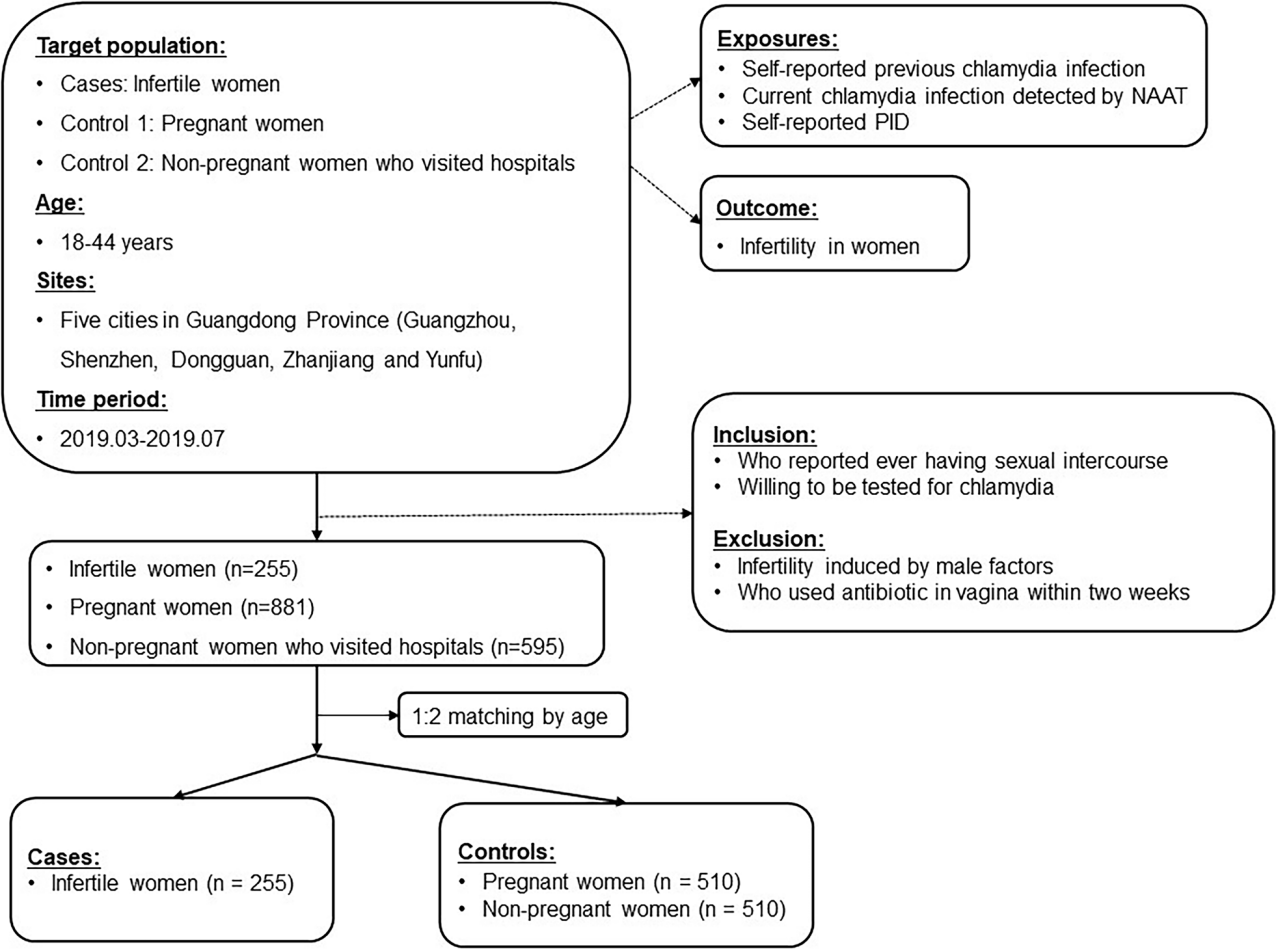
Flowchart

We performed a 1:2 matched case–control study with two control groups: pregnant controls and non-pregnant controls. For each infertility case that was ascertained, two control women were matched by age (± 3 years). For pregnant controls, we included pregnant women who were documented in the selected hospitals of the study cities. As non-pregnant controls, we selected those who sought health care other than infertility in Obstetric/Gynecologic clinics, Family Planning clinics, Dermatology & STD Department or Urological department for the first time in the past one year, and reported ever having sexual intercourse and willing to be tested for chlamydia. Indications for exclusion were the same with that of cases. Finally, we included 255 infertile women, 510 pregnant women (control 1), and 510 non-pregnant women (control 2) in this study. Information about baseline characteristics including sociodemographic characteristics, reproductive history, smoking and alcohol drinking, previous history of diseases, and sexually transmitted infection (STI) were collected through online questionnaires (Fig. 1).

The study was approved by the Institutional Review Board of Dermatology Hospital of Southern Medical University. All participants have given written informed consent.

### Sample collection and nucleic acid amplification testing

The first-stream specimen of urine samples was collected from all participants. At least one-hour interval before the last urination was needed. Self-cleaning of the urination site was not allowed. After collection, transferred it into cobas®PCR MEDIA tubes and mixed upside down five times. Then stored the sample under 2–8 °C. Cobas® 4800 (Cobas® 48000 CT/NG Amplification/Detection Kit, Shanghai, China) was used to process urine specimens for the diagnosis of *chlamydia* trachomatis.

According to the Health Industry Standard of the People's Republic of China (WS/T 513-2016), women with positive NAAT result but without any related symptoms such as odynuria, frequent and/or urgent micturition, hypogastralgia, lumbago, abnormal vaginal bleeding, and/or discharge, would be diagnosed as asymptomatic chlamydia infection. And those with positive NAAT result and related symptoms would be regarded as symptomatic chlamydia infection.

### Sample size calculation

In this matched 1:2 case–control study, we supposed the prevalence of chlamydial infection among women to be 4.7% [14], the odd ratio of chlamydial infertility to be 1.91 [15], α = 0.05, β = 0.2. Finally, a sample of 239 infertile cases was obtained, and controls were supposed to be 478 per group.

For PID, about 4.4% of sexually active women received a diagnosis of PID in their lifetime [16], and self-reported PID was associated with a fourfold higher risk of infertility [6]. Then, a sample of 36 infertile cases with matched 72 non-infertile controls can achieve 81% power under α = 0.05.

Overall, at least 239 infertile cases with matched 478 non-infertile controls were needed.

### Statistical analysis

We used the chi-square test to evaluate the baseline characteristics between non-pregnant/pregnant women and infertile women. Associations between chlamydial infection, including NAAT verified infection and self-reported previous infection, and PID with infertility in women were studied by conditional logistic regression, calculating odds ratios (ORs) with their corresponding 95% confidence intervals (CIs), adjusting for maternal age, BMI, monthly income, chronic disease, and other genital tract infection, which were suggested to be potential risk factors of infertility by published study [8]. Besides, previous chlamydial infection was added to adjust for the association between PID and infertility.

We conducted several sensitivity analyses. Firstly, we compared different effects on primary infertility and secondary infertility. The former refers to those women who have not been pregnant previously, while the latter is denoted for the inability to repeat conception after a prior pregnancy [2]. Then, considering that some of the infertility subtypes were probably not to be associated with chlamydial infection or PID, we kept infertility cases induced by tubal disorders only and excluded those related to sex factors and/or cervical/uterine/vaginal factors.

Data analyses were performed using Stata 14 software (StataCorp LLC, Texas). Statistical significance was considered at a two-sided *P* value < 0.05.

## Results

### Social demographic characteristics

Our study involved 1275 participants, including 510 non-pregnant women, 510 pregnant women, and 255 infertile women, with an average age ($${\overline{\text{x}}}$$ ± s) of 30.0 ± 4.5, 29.8 ± 4.4 and 29.8 ± 4.4 years, respectively. We compared baseline characteristics and previous history of diseases between non-pregnant/pregnant women and infertile women (Table 1). Most participants only received high school education or lower and earned less than $1500 (about 10,000 RMB) per month. And compared with control groups, more infertile women earned over $1500 per month for (*P* < 0.05). The (pregestational) BMI of infertile women was higher than that of pregnant women. For non-pregnant women, the age of sexual debut was lower than that of infertile women (*P* < 0.05). Pregnant women were more likely to have chronic diseases but less likely to experience PID and other genital tract infections in the past.

**Table 1:** Characteristics of study participants in Guangdong, China, 2019 (N = 1275)

Characteristics	Infertile women (n = 255)	Pregnant women (n = 510)	Non-pregnant women (n = 510)
	n (%)	n (%)	n (%)
Age (years, ${\overline{\text{x}}}$ ± s)	29.8 ± 4.4	29.8 ± 4.4	30.0 ± 4.5
< 25	26 (10.2)	52 (10.2)	61 (12.0)^*^
25–29	111 (43.5)	222 (43.5)	169 (33.1)
≥ 30	118 (46.3)	236 (46.3)	280 (54.9)
BMI (kg/m^2^, ${\overline{\text{x}}}$ ± s)	21.1 ± 2.7	20.6 ± 2.5^*^	20.9 ± 2.1
≤ 18.4	40 (15.7)	97 (19.0)	45 (8.8)^*^
18.5–23.9	173 (67.8)	356 (69.8)	415 (81.4)
≥ 24	42 (16.5)	57 (11.2)	50 (9.8)
Marital status			
Single	23 (9.0)	29 (5.7)	98 (19.2)^*^
Married	232 (91.0)	475 (93.1)	392 (76.9)
Other or unknown	0 (0.0)	6 (1.2)	20 (3.9)
Education			
High school or lower	153 (60.0)	286 (56.1)	321 (62.9)
Bachelor or higher	102 (40.0)	220 (43.1)	183 (35.9)
Missing	0 (0.0)	4 (0.8)	6 (1.2)
Income (dollars/year)^a^			
< 1500	179 (70.2)	440 (86.3)^*^	485 (95.1)^*^
1500–4499	46 (18.0)	57 (11.2)	11 (2.2)
≥ 4500	30 (11.8)	7 (1.4)	8 (1.6)
Missing	0 (0.0)	6 (1.2)	6 (1.2)
Smoking			
No	249 (97.6)	500 (98.0)	492 (96.5)
Yes	6 (2.4)	9 (1.8)	15 (2.9)
Missing	0 (0.0)	1 (0.2)	3 (0.6)
Alcohol			
No	216 (84.7)	444 (87.1)	418 (82.0)
Yes	39 (15.3)	56 (11.0)	81 (15.9)
Missing	0 (0.0)	10 (2.0)	11 (2.2)
Chronic condition^b^			
No	244 (95.7)	449 (88.0)^*^	479 (93.9)
Yes	11 (4.3)	61 (12.0)	31 (6.1)
Age at sexual debut (years, ${\overline{\text{x}}}$ ± s)	22.1 ± 3.7	22.5 ± 3.9	21.4 ± 3.7^*^
< 25	197 (77.3)	349 (68.4)	393 (77.1)
≥ 25	58 (22.7)	141 (27.6)	82 (16.1)
Missing	0 (0.0)	20 (3.9)	35 (6.9)
Parity			
0	168 (65.9)	159 (31.2)^*^	114 (22.4)^*^
≥ 1	87 (34.1)	351 (68.8)	394 (77.3)
Missing	0 (0.0)	0 (0.0)	2 (0.4)
Other genital tract infection^c^			
No	179 (70.2)	468 (91.8)^*^	391 (76.7)
Yes	76 (29.8)	42 (8.2)	119 (23.3)
NAAT chlamydia infection^d^			
Neg	240 (94.1)	472 (92.5)	471 (92.4)
Pos	15 (5.9)	37 (7.3)	36 (7.1)
Missing	0 (0.0)	1 (0.2)	3 (0.6)
PID^e^			
No	211 (82.7)	492 (96.5)^*^	464 (91.0)^*^
Yes	44 (17.3)	15 (2.9)	46 (9.0)
Missing	0 (0.0)	3 (0.6)	0 (0.0)
Chlamydia infection history^f^			
No	249 (97.6)	506 (99.2)^*^	508 (99.6)^*^
Yes	6 (2.4)	2 (0.4)	2 (0.4)
Missing	0 (0.0)	2 (0.4)	0 (0.0)

^a^1 dollar = 6.67 RMB

^b^Chronic disease was defined as having at least one of the following diseases previously: hepatic disease, diabetes, hyperthyroidism, hypothyroidism, or kidney disease

^c^Other genital tract infection referred to having at least one of the following diseases previously: nongonococcal urethritis, genital tract mycoplasma infection, trichomoniasis, bacterial vaginitis, gonorrhea, herpes genitalis, and HIV

^d^NAAT, nuclear acid amplification test

^e^PID, pelvic inflammatory disease, which was self-reported

^f^Chlamydia infection history was self-reported by participants

^*^There were significant differences between non-pregnant/pregnant women and infertile women, *P* < 0.05

**Fig. 2:**
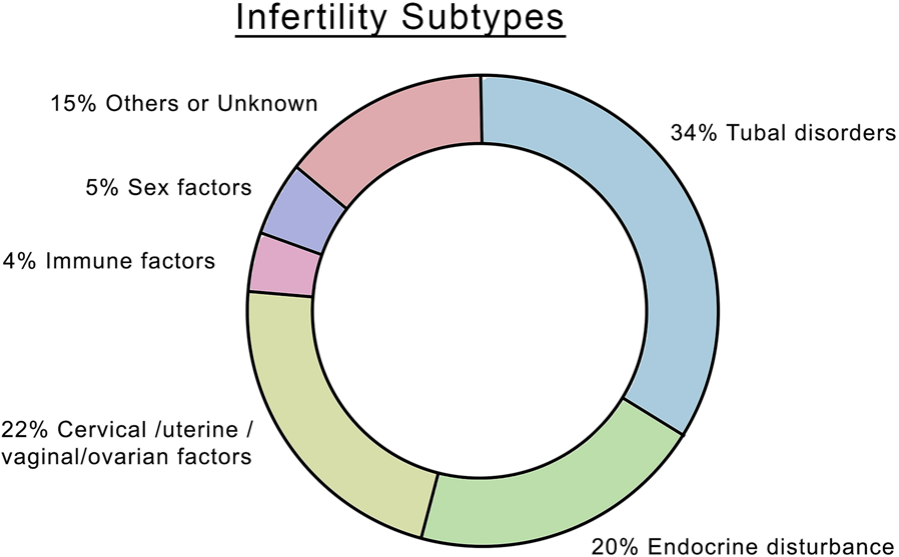
Infertility subtypes

As mentioned above, we further classified infertility into several subtypes, and Fig. 2 showed that tubal disease was an important cause of infertility, which accounted for 34% of our cases. And endocrine disturbance (20%) was also worth attention.

### Chlamydial infection

The prevalence of self-reported previous chlamydial infection was significantly higher in infertile women (2.4%) than in controls (Non-pregnancy: 0.4%; Pregnancy: 0.4%). And the current infection rates tested by NAAT were 5.9%, 7.1%, and 7.3% in infertile, non-pregnant and pregnant women, respectively. In this study, the percentage of asymptomatic infection was lower among control groups (non-pregnancy: 26.5%, pregnancy: 22.7%) than cases (46.2%).

### The association between chlamydial infection, previous PID, and infertility

Our study found that previous PID diagnoses were associated with an increased risk of infertility using non-pregnant controls (adjusted OR = 2.57, 95% CI 1.51, 4.39) and pregnant controls (adjusted OR = 6.83, 95% CI 3.47, 13.43). For current chlamydial infection, none of the odds ratios were significant at the 0.05 level (Table 2). Due to the low prevalence of self-reported chlamydia infection, it was not a valid variable for this analysis, so the result was not shown.

**Table 2:** The association between chlamydial infection, previous PID and infertility: conditional logistic regression model

Exposure	Cases: infertility										
	Cases (n = 255)	Controls: non-pregnant women (n = 510)					Controls: pregnant women (n = 510)				
	No. (%)	No. (%)	cOR (95% CI)^a^	*P*	aOR (95% CI)^b^	*P*	No. (%)	cOR (95% CI)^a^	*P*	aOR (95% CI)^b^	*P*
NAAT chlamydia infection											
No	240 (94.1)	471 (92.9)	Referent		Referent		472 (92.7)	Referent		Referent	
Yes	15 (5.9)	36 (7.1)	0.80 (0.43, 1.51)	0.50	0.56 (0.25, 1.26)	0.16	37 (7.3)	0.80 (0.43, 1.48)	0.48	0.66 (0.32, 1.38)	0.27
Previous PID diagnosis											
No	211 (82.7)	464 (91.0)	Referent		Referent		492 (97.0)	Referent		Referent	
Yes	44 (17.3)	46 (9.0)	2.07 (1.33, 3.22)	< 0.01	2.57 (1.51, 4.39)	< 0.01	15 (3.0)	6.55 (3.53, 12.19)	< 0.01	6.83 (3.47, 13.43)	< 0.01

^a^cOR, crude OR

^b^aOR, adjusted OR. For NAAT chlamydia infection, adjusted for maternal age, BMI, monthly income, chronic disease, other genital tract infection. For previous PID diagnosis, adjusted for maternal age, BMI, monthly income, chronic disease, other genital tract infection and chlamydia infection history

### Sensitivity analysis

We further evaluated the effects of PID on different subtypes of infertility. Firstly, we compared primary infertility (n = 168) and secondary infertility (n = 87). PID history was positively associated with primary infertility using non-pregnant controls (adjusted OR = 4.41, 95% CI 1.21, 16.12) and using pregnant controls (adjusted OR = 3.78, 95% CI 1.15, 12.41) (Table 3).

**Table 3:** Association between PID and infertility, stratified by parity

Exposure	Cases: primary infertility (n = 168)				Cases: secondary infertility (n = 87)			
	Controls: non-pregnant women (n = 336)		Controls: pregnant women (n = 336)		Controls: non-pregnant women (n = 174)		Controls: pregnant women (n = 174)	
	aOR (95% CI)^a^	*P*	aOR (95% CI)^a^	*P*	aOR (95% CI)^a^	*P*	aOR (95% CI)^a^	*P*
PID								
No	Referent		Referent		Referent		Referent	
Yes	4.41 (1.21, 16.12)	0.03	3.78 (1.15, 12.41)	0.03	1.91 (0.54, 6.76)	0.31	9.62 (1.70, 54.39)	0.01

^a^aOR, adjusted OR, adjusted for maternal age, BMI, monthly income, chronic disease, other genital tract infection and chlamydia infection history

Further, we kept infertility cases caused by tubal disorders (n = 86) only, and the positive association between PID and infertility was more obvious for both groups (see Additional file 1: Table S1). Previous PID was associated with an increased tubal infertility risk of 7.98-fold (95% CI 2.76, 23.06) for the non-pregnancy control group and 16.17-fold (95% CI 4.59, 56.97) for the pregnancy control group. Finally, we dropped infertility cases caused by sex factors and/or cervical/uterine/vaginal factors (n = 48), which may be less attributed to maternal infection and PID. Additional file 1: Table S2 showed that the effect size was also elevated to some extent (non-pregnant group: adjusted OR = 3.09, 95% CI 1.66, 5.76; pregnant group: adjusted OR = 7.29, 95% CI 3.37, 15.77).

## Discussion

In our evaluation of the association between chlamydial infection, PID and infertility, we found that previous PID was indicated to largely elevate the risk of infertility, especially tubal infertility. The prevalence of infertility among women with self-reported previous chlamydia infection was six times the prevalence among women with negative results, although our sample size limited an evaluation of an association of infertility with it. For current chlamydial infection, none of the odds ratios were significant at the 0.05 level. This study extended the existing literature by using two groups of controls and using NAAT methods to improve the testing accuracy.

Previous PID was indicated to elevate the risk of infertility in this study, and this finding aligned with the existing literature. A national health and nutrition examination survey conducted in the United States found that self-reported PID was associated with a fourfold higher risk of infertility among young women (18–29 years old) [6]. Another study conducted in China using a case–control study design also drawn similar conclusions [7]. Besides, the effect was stronger in pregnant women's control settings than non-pregnant control setting in our study, which may be due to the confounding of infertile cases among non-pregnant women who were unaware of their infertile facts. PID is common and approximately 4.4% sexually active women were reported to have PID [17], and its long-term harmful effects on reproductive disability put an urgent need of public health prevention measures. On the one hand, efforts can be made on identifying noninvasive PID biomarkers considering the current unspecific invasive diagnostic methods [18]. On the other hand, *Chlamydia trachomatis* is a potentially important cause of PID, and implementing related screening programs would be the most important public health measure for the prevention of PID [18].

The number of self-reported prior chlamydial infection was limited in this study, which may be due to recall bias and low detection rate in China, therefore we did not conduct the association analysis between infertility with it. When indicating prior chlamydial infections, currently available methods include self-reports and chlamydial serologic assays with poor sensitivity, leading to incomplete understanding of chlamydial PID and infertility, so it is a necessity to find an appropriate marker. It is suggested that Pgp3 antibody may better satisfy this need [16]. Our team used an ultrasensitive and high-throughput luciferase immunosorbent assay, with good specificity and sensitivity, for detection of anti-Pgp3 antibodies among reproductive-aged women, including some infertile women (unpublished). Future studies will continue for Pgp3, chlamydia, PID and infertility. From early teenagers before infection until after being a mother, a wider population level follow-up cohort study would be the most robust way to understand its natural history, although it seemed not to be feasible [19].

The association between PID and infertility should be taken in the context of the study design and other limitations beyond those already mentioned. It was unavoidable to bring recall bias when applying a case–control design. Previous PID cases, as exposures of interest, were recalled based on their past diagnosis and may be biased due to the respondents’ distorted or incomplete memory, but we combined objective records from the Hospital Information System and doctors helped the participants to finish the questionnaire. This may reduce recall bias to some extent. Then, Berkson's bias cannot be ignored when analyzing the relationship between current chlamydial infection and infertility. Recruited from hospitals, infertile women involved in this study have possibly experienced a series of examinations including chlamydia testing and further received treatment, which may explain the relatively low rate of NAAT (+) and the biased effects observed. But it was noteworthy that infertility was a long-term consequence, and it was obscure whether infection occurred before infertility among women with positive NAAT.

## Conclusions

In summary, previous PID was suggested to be associated with an elevated risk of infertility, especially tubal infertility. And for further assessment of the burden of chlamydia infertility, finding sensitive biomarkers that might predict prior chlamydial infection is urgently needed, because for most women present for infertility, inciting infection would no longer be detected by NAAT or other tests for active urogenital infection.

## Supplementary Information

**Additional file 1**. Sensitivity analysis of the association between chlamydial infection, previous PID and infertility subtypes.

## Data Availability

The data sets generated during and/or analyzed during the current study are available from the corresponding author on reasonable request.

## Abbreviations

PID: Pelvic inflammatory disease
STI: Sexually transmitted infection
NAAT: Nucleic acid amplification testing
ORs: Odds ratios
CIs: Confidence intervals
