# Supplementary material for: Chlamydia infection, PID, and infertility: further evidence from a case–control study in China
Source: BMC Womens Health. 2022 Jul 15;22:294. doi: 10.1186/s12905-022-01874-z (PMC9284834; doi:10.1186/s12905-022-01874-z)
Supplement: Supplementary file 1 — Additional file 1. Sensitivity analysis of the association between chlamydial infection, previous PID and infertility subtypes. [file 12905_2022_1874_MOESM1_ESM.docx]

# Additional file

# Tables

Table S1 The association between chlamydial infection, previous PID and infertility induced by tubal disorders

Table S2 The association between chlamydial infection, previous PID and infertility, exlcuding sex factors and cervical /uterine /peritoneal factors

**Table S1 The association between chlamydial infection, previous PID and infertility induced by tubal disorders**

| Exposure | Cases: Tubal infertility (n=86) | | | | | | | |
| --- | --- | --- | --- | --- | --- | --- | --- | --- |
|  | Controls: Non-pregnant women (n=172) | | | | Controls: Pregnant women (n=172) | | | |
|  | cOR (95% CI) ^a^ | *P*-value | aOR (95% CI) ^b^ | *P*-value | cOR (95% CI) ^a^ | *P-*value | aOR (95% CI) ^b^ | *P*-value |
| NAAT chlamydia infection |  |  |  |  |  |  |  |  |
| No | Referent | | | | Referent | | | |
| Yes | 0.87 (0.30, 2.50) | 0.79 | 0.61 (0.17, 2.21) | 0.45 | 0.91 (0.32, 2.62) | 0.86 | 0.27 (0.27, 2.85) | 0.84 |
| Previous PID diagnosis |  |  |  |  |  |  |  |  |
| No | Referent | | | | Referent | | | |
| Yes | **4.44 (2.03, 9.71)** | **<0.01** | **7.98 (2.76, 23.06)** | **<0.01** | **15.33 (4.60, 51.07)** | **<0.01** | **16.17 (4.59, 56.97)** | **<0.01** |

^a^ cOR, crude OR

^b^ aOR, adjusted OR. For NAAT chlamydia infection, adjusted for maternal age, BMI, monthly income, chronic disease, other genital tract infection. For previous PID diagnosis, adjusted for maternal age, BMI, monthly income, chronic disease, other genital tract infection and chlamydia infection history.

**Table S2 The association between chlamydial infection, previous PID and infertility, excluding sex factors and cervical /uterine /peritoneal factors**

| Exposure | Cases: Infertility, excluding sex factors and cervical /uterine /peritoneal factors (n=207) | | | | | | | |
| --- | --- | --- | --- | --- | --- | --- | --- | --- |
|  | Controls: Non-pregnant women (n=414) | | | | Controls: Pregnant women (n=414) | | | |
|  | cOR (95% CI) ^a^ | *P*-value | aOR (95% CI) ^b^ | *P*-value | cOR (95% CI) ^a^ | *P-*value | aOR (95% CI) ^b^ | *P*-value |
| NAAT chlamydia infection |  |  |  |  |  |  |  |  |
| No | Referent | | | | Referent | | | |
| Yes | 0.95 (0.49, 1.82) | 0.87 | 0.65 (0.27, 1.52) | 0.32 | 1.11 (0.59, 2.11) | 0.74 | 0.91 (0.42, 1.98) | 0.82 |
| Previous PID diagnosis |  |  |  |  |  |  |  |  |
| No | Referent | | | | Referent | | | |
| Yes | **2.04 (1.26, 3.31)** | **<0.01** | **3.09 (1.66, 5.76)** | **<0.01** | **6.92 (3.43, 13.97)** | **<0.01** | **7.29 (3.37, 15.77)** | **<0.01** |

^a^ cOR, crude OR

^b^ aOR, adjusted OR. For NAAT chlamydia infection, adjusted for maternal age, BMI, monthly income, chronic disease, other genital tract infection. For previous PID diagnosis, adjusted for maternal age, BMI, monthly income, chronic disease, other genital tract infection and chlamydia infection history.
